# Acquired subgaleal fluid collections in childhood: A case series

**DOI:** 10.1016/j.jdcr.2024.07.025

**Published:** 2024-08-10

**Authors:** Théo Brochet, Sarah Fleury, Alma Poulain, Stéphane Buchet, Yves Turc, Emmanuelle Bourrat, Eve Puzenat

**Affiliations:** a Department of Dermatology, GHPSO, Creil, France; b Department of Dermatology, CHU Amiens, Amiens, France; c Dr Buchet’s Dermatology Practice, Thonon Les Bains, France; d Department of Dermatology, Hôpital Robert Debré, APHP, Paris, France; e Department of Dermatology, CHU Besançon, Besançon, France

**Keywords:** neonatal scalp swelling, scalp swelling, subgaleal fluid collection

## Introduction

Subgaleal collections in children can be divided into 2 main etiologies: Delayed subaponeurotic (subgaleal) fluid collection (DSFC) and subgaleal hematomas. In its classic form, DSFC appears a few weeks after a cephalic birth with instrumental assistance. It forms a soft, painless, skin-colored, noninflammatory subgaleal tumefaction.^1^ When the presentation is typical, there is no need for further investigation. It regresses spontaneously within a few weeks without sequelae.

Subgaleal hematomas are more frequent, can occur at any age and are usually preceded by trauma. They can be complicated by hemorrhagic shock and reveal coagulation anomalies or intracranial vascular malformation.

This case series reports 2 cases of DSFC and 1 atypical subgaleal hematoma. DSFC is a rare or more likely under-reported cause of scalp swelling. Recognizing them and distinguishing them from other scalp swellings in infancy means that no further tests or treatments need to be carried out in typical forms.

## Case presentation

### Case 1

**Fig 1:**
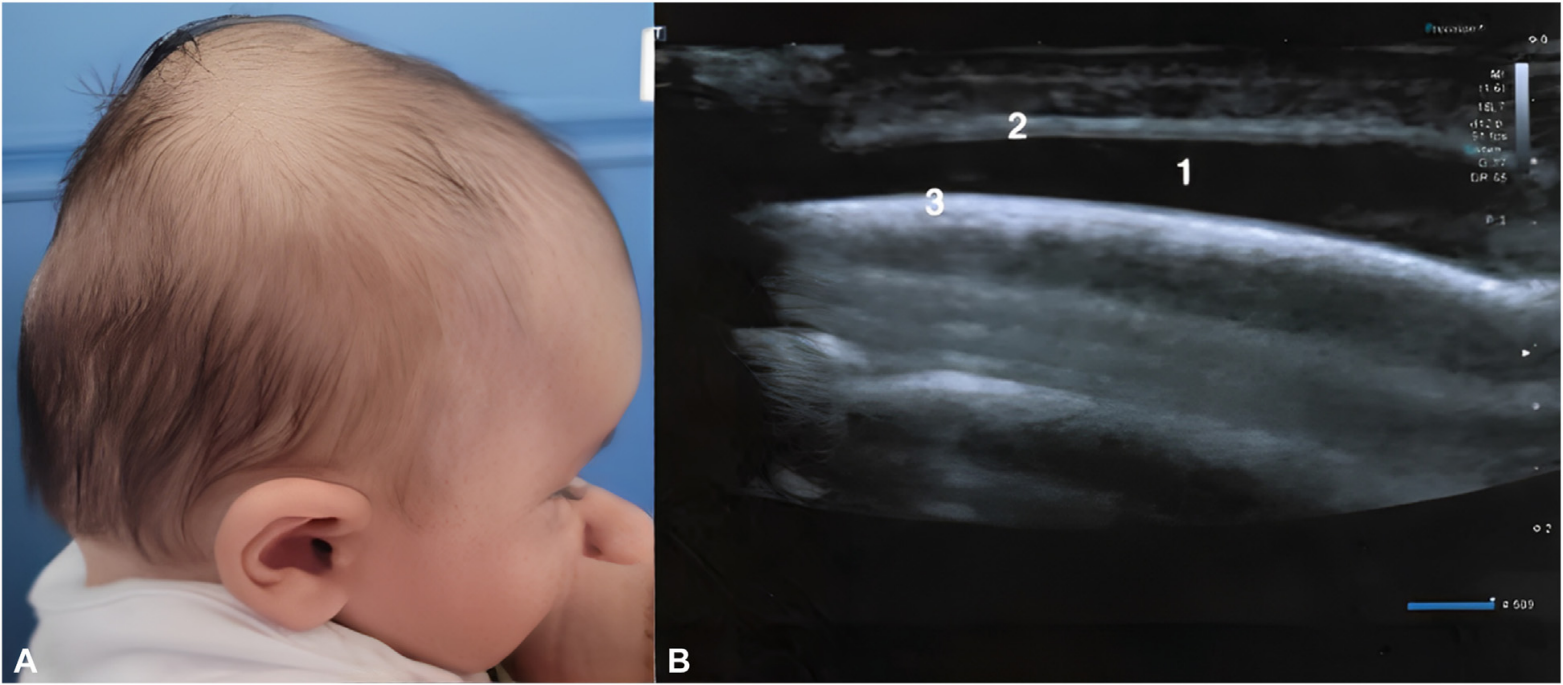
**A,** 7 × 5 centimeters parietal scalp swelling; **B,** ultrasound of this subgaleal collection (1: subaneurotic fluid collection; 2: galea aponeorotica; 3: periosteum of the parietal bone).

A four-month-old child presented with a 7 × 5 cm acquired swelling of the vertex for 1 week. This swelling is soft, stable, painless to the touch, skin-colored, and without discomfort caused by palpation (Fig 1, *A*). This patient had no medical history and was born with the aid of forceps from an uncomplicated pregnancy. There seemed to be no apparent trauma, apart from repetitive vigorous rubbing of the occiput in the decubitus position. An ultrasound scan (Fig 1, *B*) revealed a subcutaneous fluid swelling, without bleeding or bone abnormality. After consultation with dermatology, the diagnosis of DSFC was made. No puncture or other complementary examination was performed. The swelling regressed in 4 weeks, without sequela.

### Case 2

**Fig 2:**
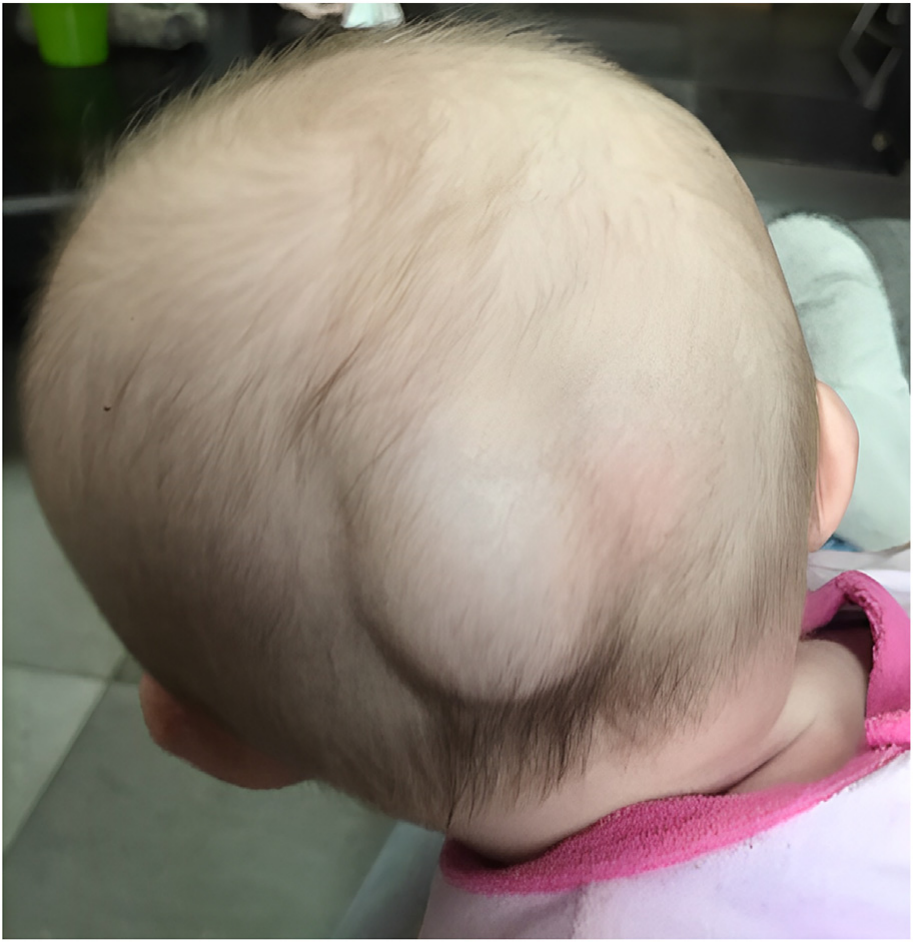
Occipital scalp swelling at the diagnosis.

A three-month-old child presented with an acquired, asymptomatic, soft, skin-colored, subcutaneous mass in the occipital region (Fig 2). This is a patient without medical history, born at term through natural delivery with the aid of suction cups and forceps. No recent head trauma was observed. An ultrasound scan showed a subcutaneous fluid swelling, nonhematic. The diagnosis of DSFC was made since the effusion was not ecchymotic, not tense, not painful, and not limited by cranial sutures. Spontaneous regression was observed within 2 months.

### Case 3

**Fig 3:**
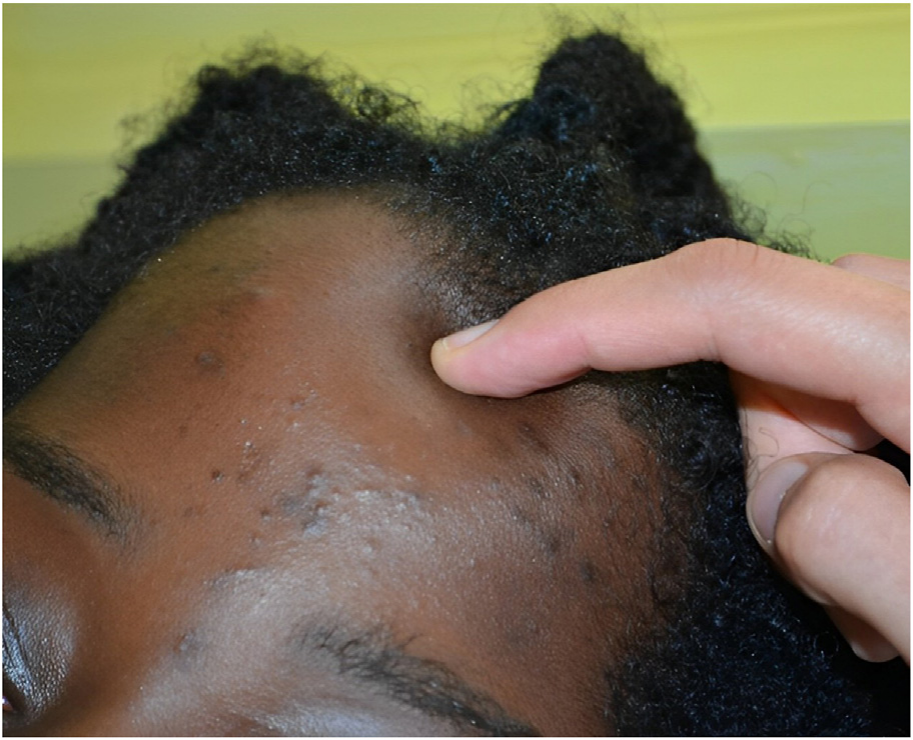
Subgaleal fronto-temporo-parietal hematoma.

**Fig 4:**
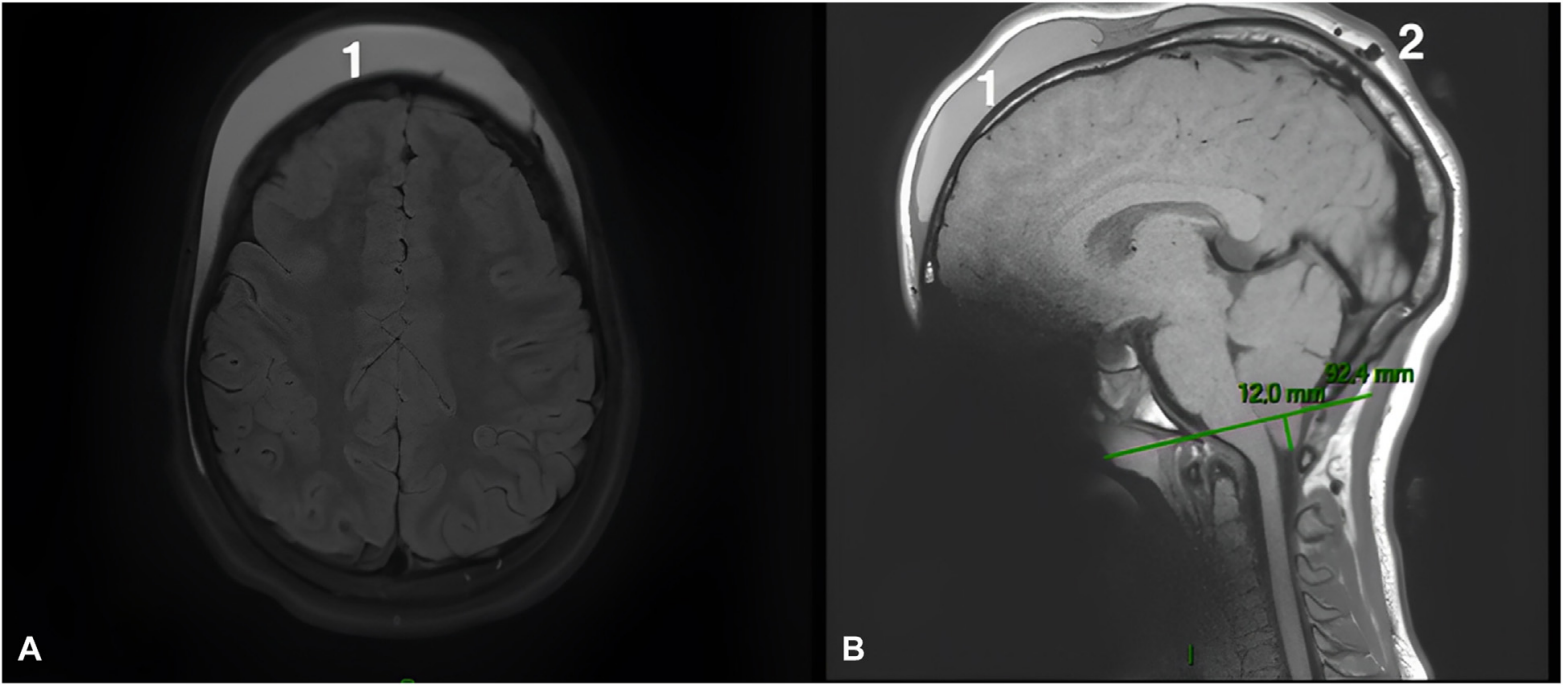
**A,** Magnetic resonance imaging frontal section T2 fat sat sequence showing this large subgaleal collection (1). **B,** Magnetic resonance imaging sagittal section T2 flair sequence showing the subgaleal collection (1) and the sinus pericranii in communication with the sagittal sinus (2).

**Fig 5:**
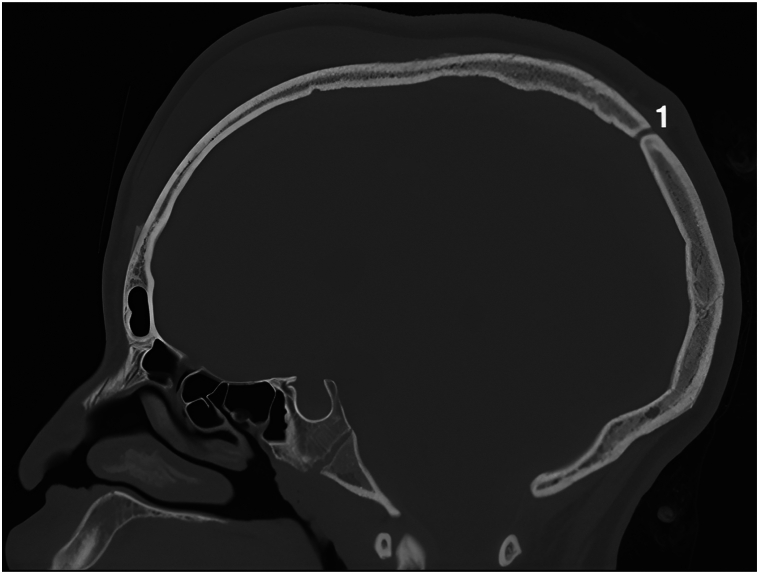
Sagittal section computerized tomography scan showing a bone defect at the two-thirds of the sagittal sinus (1).

A 13-year-old adolescent presented with an acute, spontaneous, painless, diffuse collection in the fronto-temporo-parietal region, flesh-colored, deforming the upper part of the skull and face (Fig 3). He was apyretic, in a very good general condition. He denied any recent or previous history of head trauma, except for detangling afro hair. He only complained about the cosmetic aspect. Magnetic resonance imaging showed a fronto-parietal subcutaneous collection, 1.2 cm thick and 15 cm anterior-posterior diameter (Fig 4, *A*). A computerized tomography scan confirmed that the effusion was serohematic and partially calcified, but also showed a small bony defect in the skull opposite the sagittal sinus (Fig 5). Puncture of this effusion revealed, in hindsight, a hematic fluid. A full coagulation studies was carried out, showing no coagulation abnormalities. After angio-magnetic resonance imaging, it was concluded that a subgaleal hematoma was complicating a sinus pericranii venous malformation (Fig 4, *B*). Arteriography confirmed this uncommon malformation, which consists of an abnormal communication between the intracranial and extracranial venous systems.

## Discussion

We describe here the 2 types of acquired subgaleal fluids collections for children.

The first type is DSFC: it is described as a rare entity with only 62 cases reported to date since the first description by Hopkins et al in 2002.^2^ However, in our opinion, this may be an uncommon but widely under-reported entity. It is a soft swelling diffusing like a bag of water on palpation, flesh-colored, and asymptomatic. It occurs in infants in excellent general condition, with an average age at onset of 8 weeks (from 2 to 18 weeks).^3^ There is no immediate trauma which could explain this effusion but there is a frequent history of obstetrical trauma, most often by forceps or suction.^1^^,^^3^^,^^4^ Like other subgaleal collections, it is not bounded by cranial sutures. However, DSFCs are asymptomatic and occur a few weeks after birth. Diagnosis is clinical, but in case of diagnostic doubt, ultrasound or radiography can confirm the diagnosis,^3^ as well as computerized tomography and magnetic resonance imaging scans, which are less available to infants.

No treatment is needed other than watchful waiting and observation. Natural progression is towards regression in an average span of 7 weeks (between 2 and 24 weeks)^3^ without any sequelae. Several cases have been treated with punctures yielding citrine fluid, with immediate regression. Such punctures are not recommended, however, as recurrence is frequent^3^ and given the risk of infection and bleeding from any puncture.^4^

Various etiological hypotheses have been put forward. The first, by Hopkins et al, was that subgaleal microbleeds at birth, initially masked by soft-tissue swelling, subsequently led to fluid accumulation through progressive liquefaction.^2^ The other hypothesis is that trauma during childbirth leads to the rupture of lymphatic vessels that no longer allow lymphatic drainage, resulting in subgaleal collections.^2^

Schoberer et al analyzed these collections after puncture. The presence of B2-transferrin and large quantities of B-trace-protein on the punctures performed suggested that this fluid contained cerebrospinal fluid. This led to the assumption that the collections were the result of microfractures or ruptures of the emissary or diploid veins connecting the intracranial venous system with the superficial scalp veins.^5^

**Fig 6:**
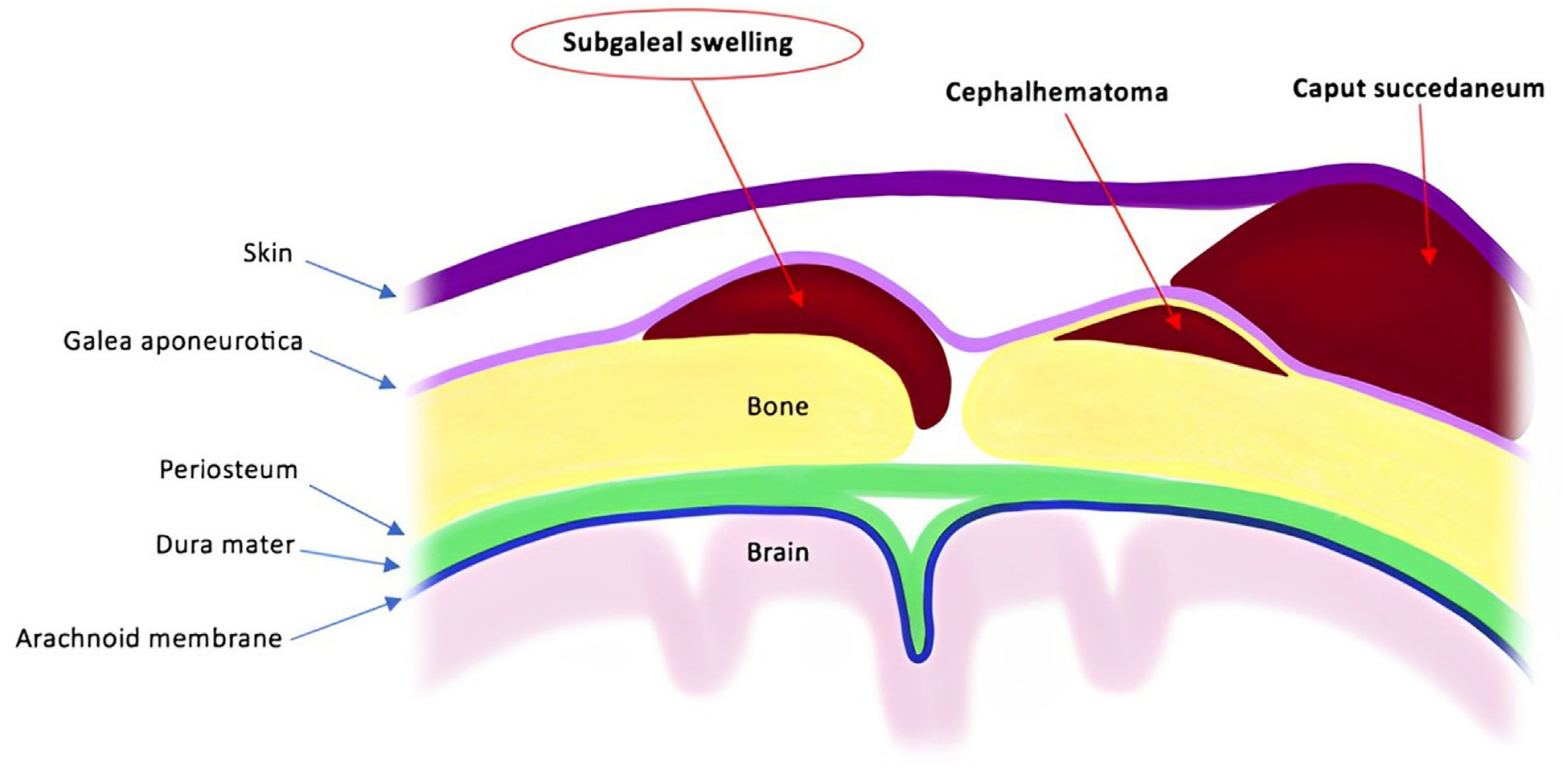
Schema of different causes of scalp swellings.

These typical cases of DSFC, which occur in the absence of recent trauma, should not obscure other causes of scalp swelling (Fig 6 and Table I). They occur after natural childbirth, are more frequent and have an earlier onset. Sometimes, they do not resolve spontaneously and require appropriate management:

- Of these, the serosanguineous bump or *caput succedaneum* is the most common. *Caput succedaneum* is an effusion between the skin and fascia that develops as a result of increased pressure on the head of a cephalically-presented newborn during labor. A few hours after birth, a bruised serosanguineous infiltration develops, which is not bounded by the cranial sutures. Spontaneous regression within a few days is typical.^6^
- Cephalohematoma is a subperiosteal collection caused by ruptured blood vessels under the periosteum, occurring in the context of assisted vacuum or forceps delivery, but also as a result of an elevated head circumference. This hemorrhage is limited by the cranial sutures, skin-colored, and can be somewhat delayed until a few hours after birth, due to slow bleeding. It resolves in 3 weeks on average, with the possibility of residual calcification disappearing a few months later.^6^
- The second type of subgaleal collection is subgaleal hematoma, which is a blood collection located between the galea and the periosteum due to ruptured veins connecting the dural sinuses and scalp veins. Like subgaleal collection, this collection is not constrained by the cranial sutures. The difference is clinical, indeed subgaleal hematoma is frequently ecchymotic, painful and taut. In neonates, this is a rare entity, but it can lead to serious neurological disorders and even death.^7^^,^^8^ Risk factors include forceps or vacuum delivery. However, subgaleal hematomas can occur spontaneously.^8^ It can cause hemorrhage of up to a third of the blood volume, leading to hypovolemic shock and/or disseminated intravascular coagulation.^7^ Onset is gradual after birth, and resolution takes 2 to 3 weeks. When treatment is required, surgical evacuation or compression bandaging of the skull is necessary, in addition to measures to prevent hypovolemic shock and disseminated intravascular coagulation.^7^

**Table I:** Main characteristics of different scalp fluid collections in childhood

	Age at onset	Localization	Color of the skin	Bounded by cranial sutures	Treatment	Time to resolution	Complications	Sequelae
Delayed subgaleal fluid collection	8 weeks on average	Between the galea and the periosteum	Skin-colored	No	Not necessary	7 weeks on average	None	None
Subgaleal hematoma	Hours to days	Between the galea and the periosteum	Ecchymotic	No	If necessary, surgical evacuation or compression bandaging	Two to 3 weeks	Hemodynamic instability or disseminated intravascular coagulation	Neurological sequelae if severe
Serosanguineous bump (*caput succedaneum*)	Minutes to hours	Between de sin and fascia	Bruise infiltration	No	Usually not necessary	Few days	Neurological deficit or hemodynamic instability if severe	None
Cephalohematoma	Hours to days	Subperiosteal	Skin-colored	Yes	Not necessary	Three to 4 weeks	Osteomyelitis	Calcification

Although subgaleal hematoma is more common in newborns, it can also occur in older children. The main causes are trauma or hair traction, as in the case presented here, these may or may not be related to coagulation anomalies or intracranial vascular malformation, but more rarely occur spontaneously.^9^^,^^10^

## Conclusion

Delayed subaponeurotic (subgaleal) fluid collection is a rare benign condition. Recognition of a typical presentation obviates the need for further investigation and results in spontaneous resolution within a few weeks. However, atypical presentations should lead to the exclusion of more worrying differential diagnoses such as subgaleal hematoma.

## Conflicts of interest

None disclosed.
